# Robust Vehicle Detection and Counting Algorithm Employing a Convolution Neural Network and Optical Flow

**DOI:** 10.3390/s19204588

**Published:** 2019-10-22

**Authors:** Ahmed Gomaa, Moataz M. Abdelwahab, Mohammed Abo-Zahhad, Tsubasa Minematsu, Rin-ichiro Taniguchi

**Affiliations:** 1 School of Electronics, Communication and Computer Engineering (ECCE), Egypt-Japan University of Science and Technology, Alexandria 21934, Egypt; moataz.abdelwahab@ejust.edu.eg (M.M.A.); mohammed.zahhad@ejust.edu.eg (M.A.-Z.); 2 Graduate School of Information Science and Electrical Engineering, Kyushu University, 744, Motooka, Nishi-ku, Fukuoka 819-0395, Japan; minematsu@limu.ait.kyushu-u.ac.jp (T.M.);; 3 National Research Institute of Astronomy and Geophysics (NRIAG), Helwan 11731, Egypt; 4 Electrical and Electronics Engineering Department, Faculty of Engineering, Assiut University, Assiut 71511, Egypt

**Keywords:** vehicle dtection, intelligent transportation system, vehicle counting, background subtraction, deep convolutional neural network

## Abstract

Automatic vehicle detection and counting are considered vital in improving traffic control and management. This work presents an effective algorithm for vehicle detection and counting in complex traffic scenes by combining both convolution neural network (CNN) and the optical flow feature tracking-based methods. In this algorithm, both the detection and tracking procedures have been linked together to get robust feature points that are updated regularly every fixed number of frames. The proposed algorithm detects moving vehicles based on a background subtraction method using CNN. Then, the vehicle’s robust features are refined and clustered by motion feature points analysis using a combined technique between KLT tracker and K-means clustering. Finally, an efficient strategy is presented using the detected and tracked points information to assign each vehicle label with its corresponding one in the vehicle’s trajectories and truly counted it. The proposed method is evaluated on videos representing challenging environments, and the experimental results showed an average detection and counting precision of 96.3% and 96.8%, respectively, which outperforms other existing approaches.

## 1. Introduction

Detecting and counting vehicles on the road are very important tasks for the traffic information analysis that can be used in traffic control and management to ensure a safe transportation system. Recently [1], vision-based vehicle detection and counting using image processing techniques provide more advantages than traditional intelligent transportation techniques [2], like microwave or magnetic detectors. Vision methods are providing high accuracy with low expenses and are better in terms of maintenance and installation. An important stage in vehicle detection and counting is the elimination of the static background from the moving objects in a challenging environment.

The most recent studies in intelligent transportation systems focus on vehicle detection [3,4,5,6,7,8,9,10,11,12]. Vehicle detection can be categorized into two groups [1]: detection methods based on vehicle appearance, and detection methods based on vehicle motion. The appearance-based strategies depend mainly on visual features, including vehicle symmetry, texture, edges, and color [5,6,7,8]. The concept of motion-based techniques is to extract the moving vehicles based on their motion characteristic that separate them from the background, such as optical flow, frame differences, and background subtraction [9,10,11]. Detection and tracking of vehicles simultaneously is considered a robust strategy for achieving accurate detection and counting results [1]. However, there are many challenges in vehicle detection and counting processes, such as illumination variation, shadows, and partial occlusion.

Yang and Qu proposed a new detection and counting technique that combined the detection and tracking processes [13]. In [13], the vehicles are detected by background subtraction with sparse and low-rank decomposition, which works with illumination or weather changes. Then, an online Kalman filter algorithm is used to track each vehicle in several frames to obtain a reliable vehicle counting result. However, this algorithm miscounts some vehicles because low-rank decomposition causes some false negative results.

In [14], a background modeling based on Principal Component Pursuit (PCP) was proposed. The training stage contains feature extraction, motion segmentation, and parameter estimation, while the counting process is based on an initial guess of vehicles number using the spatial information of a given frame, followed by a refinement process using the temporal information from previous frames. This strategy miscounts some vehicles because the counting decision depends only on the foreground detection results from the PCP that include some false positive results.

In [15], a fast strategy was presented for vehicles counting using the Gaussian Mixture Modeling (GMM) for a small area of the frame to model the background and extract the foreground vehicles. Then, a new counting strategy is applied when vehicles are passing this area. However, this method depends on a traditional background modeling method that cannot efficiently work with the different weather condition and in night scenes.

Most vehicle counting strategies depend mainly on background modeling with good accuracy, yet some false positive and false negative results have occurred when the background has large changes. Recently, the convolution neural networks have been used in object detection with better foreground and background discrimination because of their powerful capabilities in extracting low, mid, and high-level image features. Better foreground detection by CNN can improve tracking and counting accuracy. CNN can demonstrate excellent performance, but the performance is not perfect. The tracking and counting accuracy can be enhanced by reducing false-positive and false-negative results using a refining process to improve the foreground detection by CNN. Thus, a new framework for vehicle counting is suggested in this paper based on the collaboration process between collected detection information using convolutional neural networks and tracking information using optical flow. The work depends on the feature point’s information analysis between the fixed number of frames, and using the temporal information of the detection and tracking feature points between the framesets to achieve better counting decision. Table 1 summarizes the motivation of our work compared with existing state-of-the-art methods.

**Figure 1:**
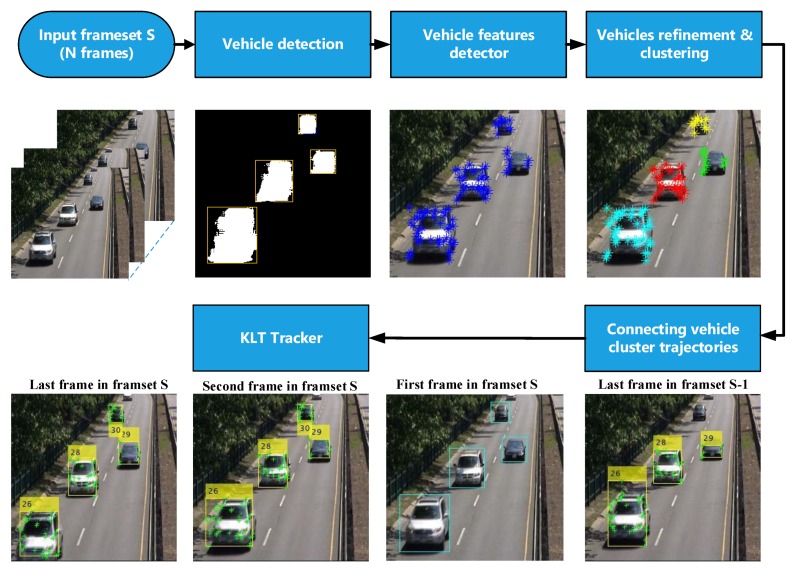
Block diagram of the proposed approach for a specific frameset *S*.

In this work, a new detection and counting method is proposed by developing a three-step approach in each frameset, as explained in Figure 1. First, the power of convolution neural network is exploited in the vehicle detection process before the vehicle refining and clustering process in the second step using the optical flow and k-means clustering. The CNN is used in the first frame, with the refining analysis considering the remaining frames in the frameset. Thus, a robust discrimination process between the foreground vehicles and noisy background regions is utilized. Thirdly, an effective counting strategy is offered to assign each vehicle with its corresponding trajectory based on the collected detection and tracking information. The detection and counting accuracy is increased, and the algorithm works efficiently with different and challenging environments. We conducted experiments on challenging datasets, and the proposed method showed the best performance in terms of precision and recall.

## 2. Methodology

The proposed framework contains three functional steps in each frameset, as shown in Figure 1. The main steps of proposed vehicle detection and counting scheme are described in the following subsections.

### 2.1. Vehicle Detection

Recently [3,4,16,17], convolutional neural networks (ConvNets) have presented excellent results in different vision challenges where it has shown an attractive characteristic to learn deep and hierarchical features, which make it more powerful than classical methods. In this work, two convolution layers, two max-pooling layers, and two fully connected feed-forward layers are adopted with the same network architecture in [16], which obtained better detection results by discriminating the foreground and background regions. The kernel size of the two convolutional layers is 5×5, and the stride of the two convolutional layers is 1. The number of channels in the first convolutional layer is 6, while that in the second convolutional layer is equal to 16, as mentioned in Table 2. The first fully connected layer has 120 hidden units, while the second fully connected layer measures the belonging foreground probability of each pixel in the frame and the output layer consists of a single sigmoid unit.

The network has 20,243 trainable weights that learned by back-propagation with a cross entropy error function:

(1) $$E=-\sum (t_{n}\ln\left(y_{n}\right)+\left(1-t_{n}\right)\ln\left(1-y_{n}\right)),$$

where $t_{n}=t\left(x_{n}\right)$ is a supervised signal of $x_{n}$ and $y_{n}$ is the network output of $x_{n}$.

**Figure 2:**
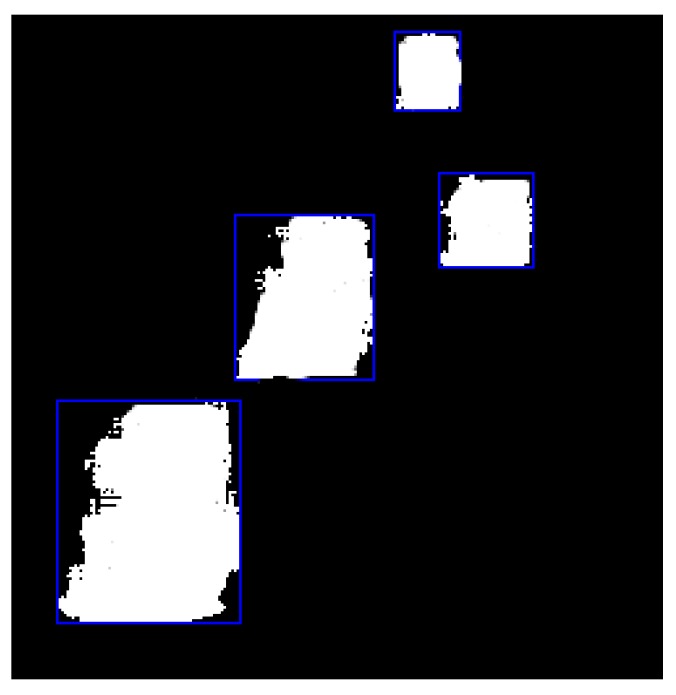
Regions of detection.

In general, the training process of CNN in the vehicle detection task requires a large annotated training set under different environments; it is highly difficult to obtain such large manually annotated dataset. In this work, we combine the segmentation result with connected component labeling and modify the training step to tackle this problem. As we focused on vehicle detection, primitive training is implemented using the foreground and background of the baseline scene in ChangeDetection.net dataset (CDnet 2014). For a different video, we need only to efficiently train the background that can be extracted automatically using the temporary median of each pixel for 150 frames of the video without changing the primitive foreground patches. The point here is to train the background efficiently, so the network can easily extract the foreground vehicles on different scenes. The detection process has been calculated in the first frame every fixed number of frames, *N* frames, as shown in Figure 2.

### 2.2. Feature Points Detector

**Figure 3:**
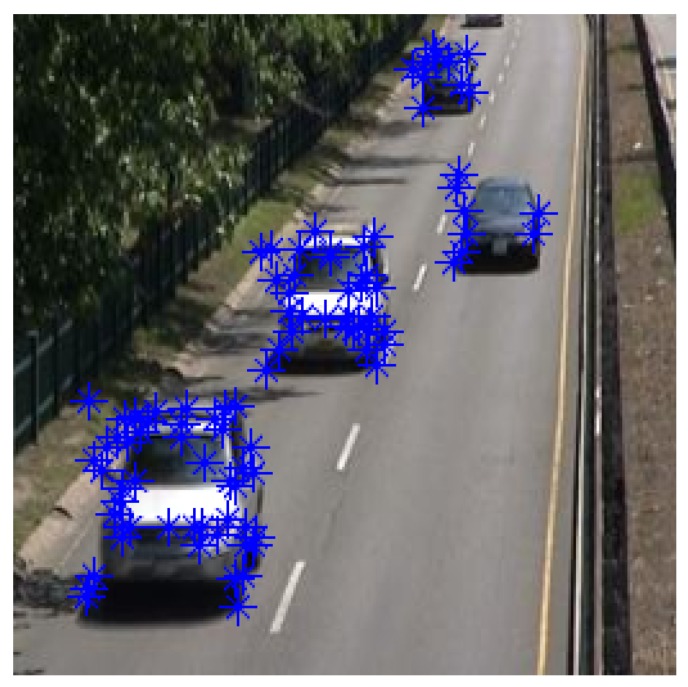
Feature points in the detected region of interest.

Selecting good features of the bounding boxes that resulted from the detection step is necessary for robustly tracking feature points across frames. While the Harris detector is the most famous corner detector, Shi and Tomasi’s detector perform better than the Harris corner detector [18]. Here, a Tomasi detector has been implemented in each detection region based on the detection algorithm described in the previous section to extract the robust corner point inside each region as illustrated in Figure 3.

The periodically updated vehicle feature points are very important to guarantee tracking them for a long time because these features may disappear as a result of illumination change and out-of-plane rotation. So, the detection process is repeated regularly every fixed number of frames, *N* frames. The new extracted feature points from the detection step and the old tracked features in the latter frame of the former *N* frames are combined. Hence, the system is updated every *N* frames by integrating both the tracking and detection feature points.

### 2.3. Vehicles Refinement and Clustering

The extracted rectangular boxes from the obtained detection result are refined and clustered to achieve better counting results by discarding the noisy part and clustering the detected vehicles. First, the feature points are extracted within the detected bounding boxes, and the optical flow-based feature point tracking is implemented for tracking the vehicle features point. The detected corner point is tracked from frame *t* to frame t+1 using the Kanade–Lucas optical flow approach [19].

The optical flow results in the first frame pairs are a set of vectors *C* e.g $C_{i}=\left(D_{i},\theta_{i}\right)$. Each element in *C* matches a feature point $P_{i}$ that tracked from frame *t* to frame t+1, where $D_{i}$ and $\theta_{i}$ are two vectors comprising the displacement magnitudes and angles respectively for each feature point, given by

(2) $$D_{i}=\sqrt{{\left(X_{2}-X_{1}\right)}^{2}+{\left(Y_{2}-Y_{1}\right)}^{2}}$$

(3) $$\theta_{i}=\arctan\left(\frac{Y_{2}-Y_{1}}{X_{2}-X_{1}}\right)$$

The noisy detections tend to result in short-lived trackers [13]. In this work, the foreground detection in the first frame is considered a vehicle object only if it is tracked in the remaining N-1 frames in the frameset.

**Figure 4:**
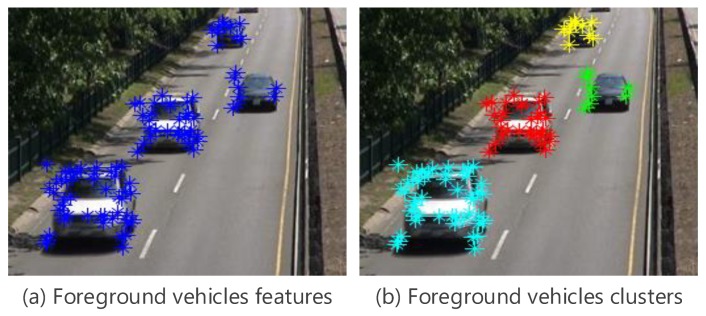
Clustering the foreground vehicles.

After refining the detection result, the number of detected regions will only contain the foreground vehicle features as shown in Figure 4a, which can be grouped based on k-means clustering as shown in Figure 4b. In this case, the elements of the vector $C_{F}$ will contain $D_{F}$, $\theta_{F}$, and points coordinates where *F* relates to the foreground. *K* value is the number of detected regions. Each feature point is a vertex $P_{i}$ = ($X_{i}$,$Y_{i}$,$D_{i}$,$\theta_{i}$), where $X_{i}$ and $Y_{i}$ represent the *X* and *Y* coordinates in the current frame, $D_{i}$ and $\theta_{i}$ represent the displacement and angle of $P_{i}$ from two consecutively frames, respectively. The vehicles corner points are extracted and clustered in the first frame of each frameset. Then KLT tracker is used to track them through the remaining frames starting from the second frame to the *N*th frame.

#### K-Means Clustering

K-means clustering is an unsupervised learning, computationally efficient algorithm for large datasets. Initially, k samples, serving as the initial centroids, are chosen randomly to approximate the centroids of the initial clusters (K is a positive integer number). Simply, K-means clustering is an algorithm to group the objects based on features into a K number of groups. The grouping process is done by minimizing the sum of squares of distances (Euclidean squared distance) between data and the corresponding cluster centroid. The K-means algorithm will be carried out by executing the following three steps below until convergence (Iterate until stable) is obtained.

- Determine the centroid coordinates.
- Determine the distance between the centroids and each data feature.
- Group the data based on the minimum distance to find the closest centroid.

In this work, K-means clustering is used after vehicle detection based on a convolutional neural network and vehicle refinement by the optical flow information in the first frame of each frameset. K-means clustering helps to achieve better counting results by grouping the vehicle features points based on their displacement, angle, and coordinates. Good detection results using CNN and vehicle detection refinement using the optical flow information give us the correct value of K that is used in the K-means algorithm. The selection process of the K value is an important issue to improve the vehicle clustering process for achieving perfect counting performance. This clustering information in the current frameset will be used with the last frame information in the previous frameset to check the connecting trajectory and make the counting decision. K value is evaluated in the first frame of each frameset and considered the value of the detected vehicles after refining the detection result (the number of refined bounding boxes).

### 2.4. Connecting Vehicle Cluster Trajectories

**Figure 5:**
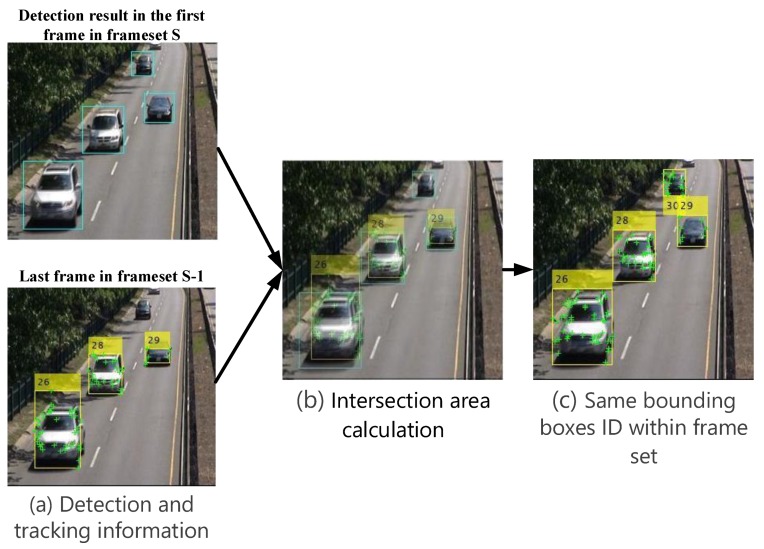
Connecting vehicles trajectories.

The detection vehicle regions with their most robust feature points inside each cluster will be accompanied by a bounding box created according to feature points’ coordinates in each cluster. Each one of the bounding boxes with their feature points will take a unique ID that tracked within the frameset as shown in Figure 5c. The intersection area between the vehicle bounding boxes in two specific consecutive frames is calculated as shown in Figure 5b; the bounding boxes for the first frame in frameset *S* and the bounding boxes for the last frame in frameset S-1 as shown in Figure 5a.

This step to judge the possibility of assigning the same ID for the new detected vehicle or it is a new vehicle with a different ID. For this purpose, the following two cases are considered.

1. Maintaining the same vehicle cluster

In this case, the intersection area is greater than a predetermined α percentage, hence the new detected vehicle has the same label (ID) of the old matched one as shown in Figure 5b, like vehicles number 26, 28, and 29. Another case of maintaining the same ID, when the detection algorithm cannot detect the tracked vehicle, then the result of the tracking will be used with the same ID for the next frameset.

2. Creating a new vehicle cluster

This case established when there is no intersection area or the estimated intersection area is less than or equal to α, so the new detected vehicle assigns a new label ID, such vehicle number 30 as shown in Figure 5b.

**Figure 6:**
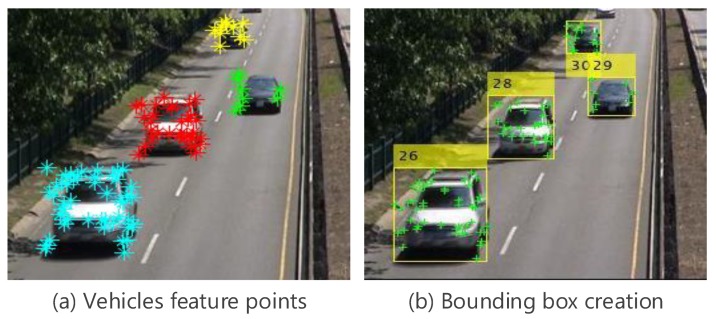
Variable bounding box.

**Figure 7:**
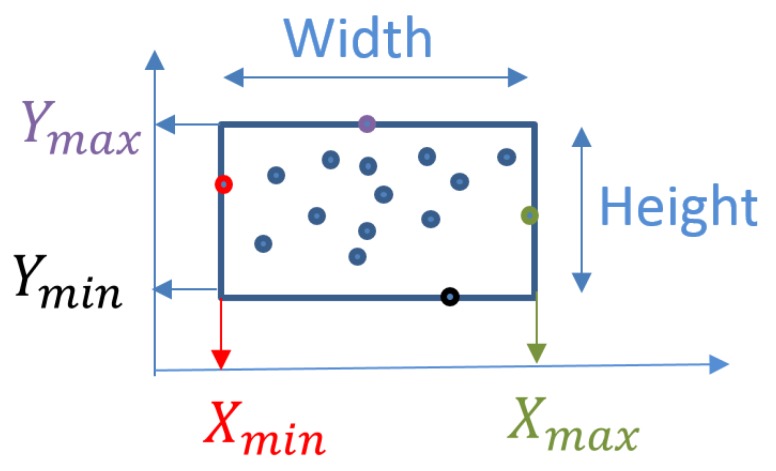
Varying bounding box coordinates.

To solve the problem of fixed bounding box size, we propose an adaptive bounding box using the output points from the optical flow, every frame after getting the strongest points as shown in Figure 6a, we can easily form the correct bounding box in relative with these points as shown in Figure 6b. Hence, for every frame after getting the strongest points, we can easily form the correct bounding box in relative with these points. This process is executed based on the minimum and maximum feature point coordinates $X_{min}$, $Y_{min}$, $X_{max}$, and $Y_{max}$ for each vehicle, as shown in Figure 7

$$Bounding\;Box=\left[X_{min}\;Y_{min}\;\left(X_{max}-X_{min}\right)\;\left(Y_{max}-Y_{min}\right)\right].$$

## 3. Experimental Results

The proposed method for detection and counting is evaluated and compared with four state of the art algorithms [12,13,14,15]. Two experiments, including seven videos with various challenges [20], are used to validate the contribution of the proposed method. We test the proposed approach on nighttime, daytime, intermittent vehicle motion, and crowd scenes, as mentioned in Table 3. In all experiments, the fixed number of frames in each frameset is equal to ten frames, *N* = 10 for achieving better tracking and counting result using KLT [21]. We examined the algorithm with different values of α, where we found that if α is too high, the same vehicle may be classified into a new vehicle. The value of α=25% yields to the best accuracy in our experiment.

The proposed method offers a robust multi-vehicles detection and counting system.

Quantitative evaluation of the detection and counting will be discussed and compared with recent approaches [12,13,14,15] to examine the detection and counting performance of the proposed methodology.

The detection accuracy is evaluated using quantitative performance metrics that have been used as a standard evaluation [22], known as Precision and Recall. The precision is calculated as the percentage of correctly detection vehicle pixels [true positive (TP)] over the total number of detecting object pixels including TPs and false positive (FP).

(4) $$Precision=\frac{\left|TP\right|}{\left|TP\right|+\left|FP\right|}$$

Recall refers to the ratio of accurately detected vehicle pixels to the number of actual vehicle pixels that include the number of false negative pixels (FN).

(5) $$Recall=\frac{\left|TP\right|}{\left|TP\right|+\left|FN\right|}$$

The counting precision can be defined as

(6) $$Precision\left(\%\right)=100-Error\left(\%\right)$$

where

(7) $$Error\left(\%\right)=\frac{\left|Estimated-TrueNo.\right|}{TrueNo.}\times 100$$

In the sake of comparison with the recent counting techniques, we assume focusing on data sets containing vehicle objects only because the counting strategies based on background subtraction technique cannot discriminate between the vehicles and personal objects. The first experiment focused on two sequences from GRAM dataset [23], M-30 and M-30 HD and HighwayII video from ATON Testbed. Comparison with recent techniques has been conducted as illustrated in Table 4 when the counting precision is used as a performance merits [15]. The proposed method achieved the highest accuracy without missing any vehicle.

In the second experiment, four video sequences from CDnet2014 dataset [24] are used to evaluate the detection and counting results of the proposed algorithm. It is asserted that the average precision rate of 96.3% for CDnet2014 dataset achieved from the proposed approach as shown in Table 5 is highly encouraging compared to 69.7%, 89.4% from the methods presented in [12,13], respectively.

The counting precision results for approaches focused on the detection and counting are reported in Table 4 and Table 6. The counting algorithm based on a combined strategy between low-rank decomposition and Kalman filter [13] showed good overall accuracy. However, this method has a declined counting precision accuracy as a result of the failure in Kalman filtering. Also, the recall dramatically drops using the low-rank decomposition detector that tends to fail in more complex scenes with false-negative results.

Although the results of [15] showed good counting precision accuracy in some videos such as GRAM dataset and ATON Testbed, the precision dramatically drops using the traditional background modeling method, Gaussian Mixture Modeling (GMM) that cannot efficiently work in different environments such as CDnet 2014 videos. The proposed algorithm achieves better counting precision as shown in Table 6, with an average counting precision percentage of 96.8% with a higher precision percentage. This result is based on the false positive detections elimination process that yields collecting information from the perfect detection step and feature points motion analysis.

Estimating the number of vehicles is the main target for traffic information analysis. The proposed approach was tested on different scenes, including night time, day time, intermittent vehicle motion, and crowded. Although the suggested strategy achieved a sufficient counting accuracy, it depends on the training process in the detection part, which tends to cause time delay in real applications. Another concern in our work is the vehicles occlusion that occurs in overlapped vehicles regions cases.

In this work, we focused on achieving accurate vehicle detection and counting. Besides, the strategy relies on multi-vehicle tracking based on optical flow to track the moving vehicles and counting them based on the detection and tracking results. The proposed algorithm may cause vehicle mis-counting in some scenes with heavy occlusions, such as tram station scene when two vehicles occluded for a long time. The congestion and intensive traffic situations in urban traffic situations often make more obstacles for the proposed approach in achieving accurate vehicle detection, tracking, and counting.

**Figure 8:**
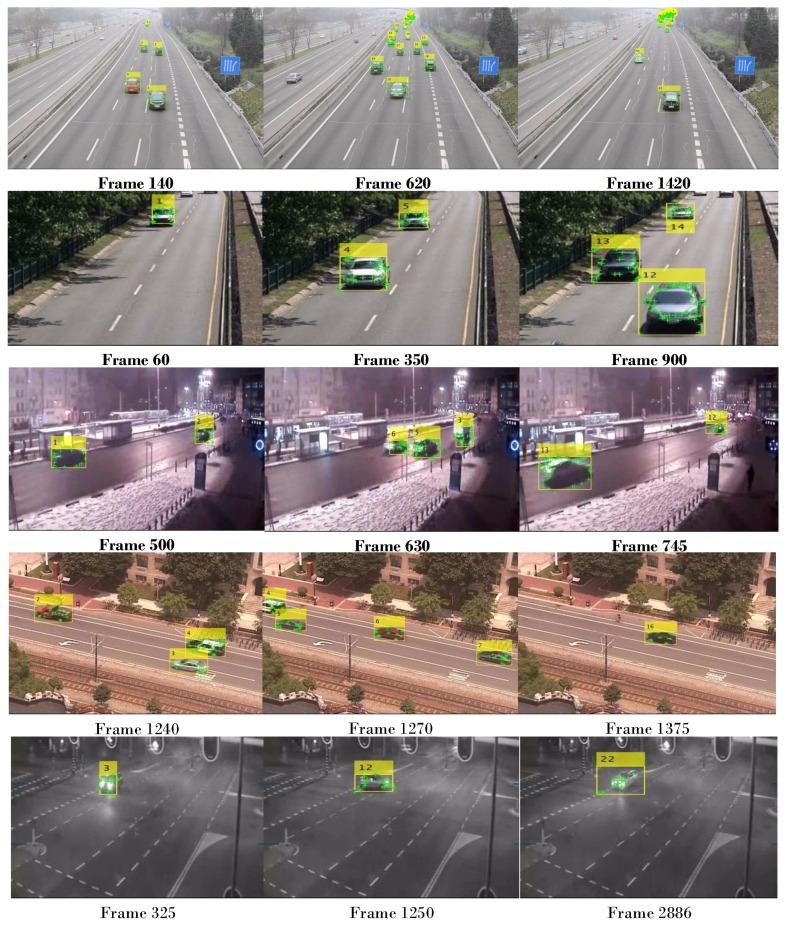
Sample results on GRAM and CDnet2014 dataset. Top row: Cloudy and crowded. Second and fourth row: Waving trees. Third and Bottom row: Night scene with changed light.

Figure 8 shows visual evaluation samples of the detection and counting results in various challenging scenes such as sunny, night, crowded, and waving trees scenes. The first, second, and fourth rows illustrate the success in handling detection and counting of vehicles in daytime highway sequences. The main problem in these videos is that the background regions, including highway and trees, are occluded by waving trees and moving car shadow. Hence, false-positive results occurred with low detection and counting accuracy. The challenge of the third row Tram station sequence is that the vehicle headlights have an intensive effect on foreground vehicle detection that results in some false-positive results. Moreover, the minimum illumination degree makes foreground vehicle detection so difficult leading to false-negative results. The main challenge of the fifth-row Street corner at night sequence is the light reflection that makes the foreground detection easily affected by the noise with false-positive and false-negative results. The third and last rows demonstrate the behavior of the proposed algorithm in night videos dealing with various dynamic light changing.

## 4. Conclusions

In this work, a new and robust vehicle detection and counting approach was proposed by developing a three-step approach. The proposed method first detects the vehicles using a CNN-based classifier with connected component labeling, then vehicle feature motion is analyzed to remove the noise and cluster the vehicles. Finally, a way to assign the detected vehicles with its corresponding cluster is introduced, to ensure a non-repeated counting process, by considering the intersection area between the detected and tracked point information. Experimental results on different datasets showed that the proposed strategy outperforms other existing methods. 

## Figures and Tables

**Table 1:** Evaluation summary with the state of art.

Compared Algorithm	Advantages	Disadvantages
Corola [12]	Low computational complexity. Good detection result.	Only detection method. Low detection result in severe illumination changes.
Yang et al. [13]	Low computational complexity. Good detection and counting accuracy. Detection and Counting algorithm.	Declined counting precision accuracy. Dropping in the recall detection accuracy.
Quesada et al. [14]	Low computational complexity. simple counting strategy with good accuracy.	Miscounts some vehicles because of some false-positive results. Counting is based on an initial guess that leads to insufficient counting accuracy. Only counting method.
Mohamed [15]	Faster counting strategy.	Cannot efficiently work with different environment and weather condition. Dropping in the overall precision accuracy by using a traditional background subtraction method. Only counting method.
Proposed Method	Low computational complexity. Better detection and counting accuracy. Detection and counting algorithm. Working with different and complex traffic scenes.	Vehicle occlusion in specific videos influence the vehicle detection and counting.

**Table 2:** The structure of the adopted convolutional neural network, where K is the size of the kernel, S is the stride [16].

Layer Type	Parameters
Input	27×27×2 pixels ( gray scale image patches )
Convolution	6 filters, K:5×5, S:1
Activate function	ReLU
Maxpooling	K:3×3, S:3
Convolution	16 filters, K:5×5, S:1
Activate function	ReLU
Maxpooling	K:3×3, S:3
Fully connected	120 hidden units
Sigmoid	2 classes ( Foreground/Background )

**Table 3:** Challenge environments information of the sequences used in the performance evaluation.

Dataset	GRAM Dataset		CDnet2014				ATON Testbed
Sequence	M-30	M-30-HD	Highway	Intermittenpan	Streetcorneratnight	Tramstation	Highway II
Challenging description	Sunny day, Low resolution camera.	High resolution camera.	Sunny day, Shadows and waving trees.	Sunny day, Waving trees.	Light changes, Night scene.	Night scene, Light changes.	Crowed scene.

**Table 4:** Vehicle counting accuracy for first experiment.

Compared Algorithm	GRAM Dataset				ATON Testbed	
	M-30		M-30-HD		Highway II	
	Miss Detection	Precision	Miss Detection	Precision	Miss Detection	Precision
Yang et al. [13]	6	92.20	5	88.10	3	92.31
Quesada et al. [14]	2	97.41	3	92.86	N/A	N/A
Mohamed [15]	1	98.70	0	100	2	95.65
Proposed Method	0	100	0	100	1	97.9

**Table 5:** Vehicle detection results comparison on CDnet2014 sequences.

Method		Corola [12]	Yang et al. [13]	Proposed Method
Sequence Videos				
Highway	Precission %	95.1	91.3	98.2
	Recall %	85.4	92.1	99.2
Intermittenpan	Precission %	56.6	90.2	98.7
	Recall %	58.5	98.8	97.5
Streetcorneratnight	Precission %	82.4	89.1	95.8
	Recall %	86.5	97.2	97.1
Tramstation	Precission %	44.7	87.1	92.3
	Recall %	91.0	97.1	97.1
Average accuracy	Precission %	69.7	89.4	96.3
	Recall %	80.4	96.3	97.7

**Table 6:** Vehicle counting accuracy for CDnet2014 sequences.

Compared Algorithm	Highway Precision	Intermittenpan Precision	Streetcorneratnight Precision	TramStation Precision
Yang et al. [13]	93.3	93.3	90.4	84.6
Mohamed [15]	92.3	N/A	N/A	N/A
Proposed Method	100	93.3	95.2	91.6
